# Cow's Milk Allergy Is a Major Contributor in Recurrent Perianal Dermatitis of Infants

**DOI:** 10.5402/2012/408769

**Published:** 2012-09-03

**Authors:** Mostafa Abdel-Aziz El-Hodhod, Ahmad Mohamed Hamdy, Marwa Talaat El-Deeb, Mohamed O. Elmaraghy

**Affiliations:** ^1^Department of Pediatrics, Faculty of Medicine, Ain Shams University, Abbassia, Cairo 11566, Egypt; ^2^Department of Clinical Pathology, Faculty of Medicine, Ain Shams University, Abbassia, Cairo 11566, Egypt

## Abstract

*Background*. Recurrent perianal inflammation has great etiologic diversity. A possible cause is cow's milk allergy (CMA). The aim was to assess the magnitude of this cause. *Subjects and Methods*. This follow up clinical study was carried out on 63 infants with perianal dermatitis of more than 3 weeks with history of recurrence. Definitive diagnosis was made for each infant through medical history taking, clinical examination and investigations including stool analysis and culture, stool pH and reducing substances, perianal swab for different cultures and staining for *Candida albicans*. Complete blood count and quantitative determination of cow's milk-specific serum IgE concentration were done for all patients. CMA was confirmed through an open withdrawal-rechallenge procedure. Serum immunoglobulins and CD markers as well as gastrointestinal endoscopies were done for some patients. *Results*. Causes of perianal dermatitis included CMA (47.6%), bacterial dermatitis (17.46%), moniliasis (15.87%), enterobiasis (9.52%) and lactose intolerance (9.5%). Predictors of CMA included presence of bloody and/or mucoid stool, other atopic manifestations, anal fissures, or recurrent vomiting. *Conclusion*. We can conclude that cow's milk allergy is a common cause of recurrent perianal dermatitis. Mucoid or bloody stool, anal fissures or ulcers, vomiting and atopic manifestations can predict this etiology.

## 1. Introduction

Perianal dermatitis is probably the most common cutaneous disorder of the genitoanal area [1]. Diaper dermatitis is observed most frequently in infants at 9–12 months of age [2]. It has a multifactorial etiology and high chronicity [3, 4]. Its prevalence is not greatly different between genders or among races [5]. Signs of diaper dermatitis including erosions have been noted as early as the first 4 days of life [6–8].

There have been only a few studies on the etiology and causative factors in anal eczema [9–11]. The patient's diet may be a factor in the development of diaper dermatitis [12]. Breastfed infants are less likely to develop moderate to severe diaper dermatitis relative to formula-fed infants [2, 13]. Adverse reactions to cow's milk are frequent (2–7%) in the first year of life and may include cutaneous (50–60%), gastrointestinal (50–60%), or respiratory (20–30%) affection [14]. Streptococcal perianal infections were reported as a frequent cause of such a recurrent condition [15].

The aim of this work was to find out the different causes of recurrent perianal dermatitis with focus on the magnitude of cow's milk allergy and the possible clinical or laboratory predictors of this etiology. 

## 2. Subjects and Methods

This follow-up clinical study was carried out on 63 infants with perianal dermatitis that persisted for more than 3 weeks and with history of recurrence. They represented 5.16% of 1220 patients presenting with the main complaint of perianal inflammation. They were seen among 42234 infants presenting to the Out-Patient Clinics, Children's Hospital, Ain Shams University (2.89%). They were 33 males and 30 females. All of them received different systemic and local remedies for the dermatitis including steroids, antifungal, and/or soothing agents prior to presentation with recurrence of their problem after either full or partial response. The study was conducted between January 2009 and December 2010. Study group was diagnosed and followed up in the Pediatric Gastroenterology Unit, Children's Hospital, Ain Shams University. 

Inclusion criteria:

1. age between 1–24 months,
2. perianal erythema for more than 3 weeks,
3. recurrent nature (more than 2 occasions).

Exclusion criteria:

1. surgical anorectal procedures,
2. napkin dermatitis not reaching the anal verge.

After approval of the ethics committee, the informed consents were taken from parents or guardians with explanation of the study and its procedures.

Each patient was subjected to the following.

(1) Medical history taking with special emphasis on description of perianal lesions regarding age of onset, duration of last attack, recurrence rate. Associated gastrointestinal symptoms included vomiting, constipation defined according to Hyams et al., [16], diarrhea defined according to Gishan [17], abdominal distension, presence of blood or mucus in the stools by naked eyes. History of recurrent infections, atopic features in the patients, and family history of erythema, atopy, and immune deficiency were also included. 

(2) Careful clinical examination was conducted including weight and length (that were plotted against growth charts), description of perianal erythema (diameter in centimeters from anal verge to the farthest outer point not including satellites, presence of satellites, ulcers or anal fissures). 

(3) Laboratory investigations at presentation include the following.

- Stool analysis for microscopic pus cells, red blood cells, and stool culture on aerobic and anaerobic media. Stool for culture as obtained through an anal swab to avoid contamination from the perianal area.
- Stool pH and reducing substances.
- Perianal swab from the erythematous lesion with culture and sensitivity with staining for *Candida albicans. *
- Complete blood count (CBC) (By Coulter 1660) and ESR.
- Serum Immunoglobulins A, G, and M
- CD markers 3, 4, and 8 when indicated in lymphopenic patients.

(4) Quantitative determination of allergen specific IgE concentration in serum against whole cow milk protein [18] was performed by enzyme-allergosorbent-test (DR-Fooke, laboratories GmbH, Mainstrable 85). 

(5) Colonoscopy and esophagogastroduodenoscopy in cases suspected of having Crohn's disease in presence of significant growth failure (weight and length below 5th centile for age) mucoid and/or bloody diarrhea or elevated ESR.

Diagnosis of cow milk allergy (CMA) was based on the results of withdrawal rechallenge [19]. Before getting the test results, cow's milk elimination diet was instituted to all patients and their lactating mothers (if breast fed) for 4 weeks with clinical assessment of erythema and other clinical features. An open rechallenge was started and patients were followed up for another 4 weeks with assessment of recurrence of dermatitis and other clinical features. The list of foods and food ingredients that are avoided were described according to Zeiger et al., [20]. In infants below one year of age, a hypoallergic amino-acid-based formula was prescribed when breast feeding was not possible. 

Diagnosis of candidal infection was based on Dixon et al. [21] and Taschdjian et al., [22]. Diagnosis of small bowel bacterial overgrowth (SBBO) was based on Ghoshal et al. [23]. Diagnosis of lactose intolerance was done through breath hydrogen test and stool pH and reducing substances [24, 25].

## 3. Statistical Analysis

The collected data were analysed using Statistical Package for Social Science (SPSS 15.0.1 for windows; SPSS Inc, Chicago, IL, 2001). Student's *t*-test was used to compare mean values of different groups and *X* ^2^ test was used to compare frequency of qualitative variables of different groups. Regression analysis was used to find out the most important predictive parameters for the diagnosis of underlying cow's milk allergy.

## 4. Results

The present study was conducted on 63 infants with a mean age of 15.01 ± 3.26 months. They were 33 males and 30 females. 

Duration of the last attack ranged between 12 and 30 days with a mean of 20.1 ± 3.5 days. Number of recurrences ranged between 2 and 6 with a mean of 3.6 ± 1.3 attacks. Age at onset of erythema ranged between 4 and 20 months with a mean of 11.2 ± 3.7 months. There was a positive history of vomiting in 33 patients (52.4 %), diarrhea in 30 patients (47.6%), constipation in 4 patients (6.3%), and abdominal distension in 27 patients (42.8%). Macroscopic assessment of stool showed blood in 32 patients (50.8%), pus in 40 patients (63.5%), and mucus in 42 patients (66.7%). Atopic features were present in 25 patients (39.7%), family history of erythema in 19 patients (30.1%), and family history of atopy in 23 patients (36.5%). 

On examination of the perianal area, extent of erythema from anal verge ranged between 1.5 and 6 cm with a mean of 3.6 ± 1.2 cm. Ulcers were present in 42 patients (66.7%), satellites in 20 patients (31.7%), and anal fissures in 27 patients (42.8%).

Microscopic stool assessment showed pus cells in 30 patients (47.6%) and RBCs in 34 patients (54%). Test for reducing substances in stools was positive in 12 patients (19%). Stool culture revealed growth of pathogenic organisms in 15 patients (23.8%).

The definite causes of perianal erythema included cow's milk allergy in 30 patients 47.6%, monilial napkin dermatitis in 10 patients 15.87%, lactose intolerance in 6 patients 9.5%, bacterial dermatitis in 11 patients 17.46% (primary immune deficiency in 2 patients 3.22%, small bowel bacterial overgrowth in 3 patients 4.76% and without an evident cause in 6 cases 9.52%). Six patients (9.52%) showed *Entrobius vermicularis* infestation with full recovery of dermatitis after eradication of infestation and recovered. In the 16 patients with monilial napkin dermatitis, only 4 cases were positive for oral moniliasis. All cases with bacterial dermatitis were positive for stool culture as well. 8/11 patients had beta hemolytic streptococci (50% concordance with stool culture) and 3 patients had Klebsiella (100% concordance with stool culture)

Hygienic awareness for parents of patients with recurrent monilial napkin dermatitis led to improvement in 8/10 patients. However, 2 patients continued to have the recurrence for 6 months and improved spontaneously without a clear explanation. Patients with lactose intolerance responded immediately to oral lactase therapy and discontinued the enzyme after 6 months without recurrence. This means that pathology was secondary rather than primary. The 2 patients with severe combined immunodeficiency unfortunately died within 6 months. Patients with small bowel bacterial overgrowth responded adequately to medical therapy. Other bacterial dermatitis responded permanently to treatment with specific culture reported systemic antibiotics. 

Vomiting, abdominal distension, atopic features in patients, ulcers, anal fissures, macroscopic and microscopic pus and blood as well as absence of satellites, reducing substances and pathogenic bacteria are all significantly in favor of CMA as a cause of perianal dermatitis (Table 1).

Serum level of IgE specific to cow's milk proteins was significantly higher in CMA compared to other groups (*P* < 0.0001). Similarly stool pH was higher in CMA group compared to all other entities (*P* = 0.007). On the other hand, size of ulcers was smaller in CMA compared to others (*P* < 0.0001) (Table 2).

The multiregression analysis showed that presence of microscopic or macroscopic blood in stool, presence of other atopic manifestations, anal fissures, presence of mucus in stool and occurrence of recurrent vomiting are the most important predictors of underlying cow's milk allergy as a cause of recurrent perianal dermatitis (Table 3).

The outcome of the studied patients varied. So, tolerance was achieved 1 year after elimination of cow's milk in allergic patients with no more recurrence of dermatitis in all of them. 

## 5. Discussion

In the present work, cow's milk allergy was the most common cause of recurrent perianal inflammation (47.6%). This may be indirectly supported with the work of Doganci and Cengizlier [26] that perianal erythema was found among 58% of children with chronic constipation related to cow's milk allergy, 10.34% of whom improved on strict dietary elimination of cow's milk products in their nutrition. Intolerance of cow's milk can cause severe perianal lesions with pain on defecation and consequent constipation and that, in such cases, a diet free of cow's milk can rapidly resolve both the constipation and related disorders [27].

The protective role of breast feeding was described by Berg et al., [28], but they ascribed the effect to the lower stool pH and less irritating levels of fecal enzymes of infants who were breastfed.

The other definite causes of perianal erythema included in order bacterial dermatitis with its different underlying causes, monilial napkin dermatitis with or without oral affection, lactose intolerance regardless its cause and *Entrobius vermicularis* infestation. 

These values are different from Kränke et al., [4] who found that, among 126 patients, the primary diagnosis in 68 patients was intertrigo/candidiasis (42.9%), atopic dermatitis (6.3%), pruritus ani (5.6%), psoriasis (3.2%), skin atrophy from steroid use (2.4%), lichen sclerosus et atrophicus (*n* = 2), herpes simplex (*n* = 1), and condylomata acuminata (*n* = 1). However, they included children and adults as well as acute and protracted cases in contrast with our study where we included infants with recurrent prolonged disease only.

The high frequency of candidal dermatitis agrees with Dixon et al., [21] who found that forty-one percent of 117 cases with napkin rash compared to 1 of 68 infants with normal skin grew *Candida albicans *in culture. A correlation between the severity of primary irritant diaper dermatitis and the level of *Candida albicans *in the feces has been reported [2, 29, 30].

Candidal infection, in our work, was not significantly associated with oral moniliasis. Singalavanija and Frieden [31] found that infants with candidal diaper dermatitis may also have *Candida albicans *in the oral mucosa. Moreover, Rasmussen [32] mentioned that candidal infection is a common complication of diaper dermatitis. This may explain the higher frequency of this pathology as it is not only a primary problem but also can complicate other causes.

The lack of contact dermatitis in our study may be based on the selection of those having perianal dermatitis only. Moreover, Shin [12] thought that as a result of the immaturity of the immunologic system of infants, true allergic contact dermatitis is rare in the diaper area.

Reporting of immunodeficiency in some cases in contrast to Kränke et al. [4] may be related to involvement of recurrent prolonged cases in our study. Paller [33] mentioned that if the condition is severe and persists, it may be the presenting feature of an immunodeficiency. 

Shin [12] mentioned that perianal streptococcal disease shows sharply demarcated, bright erythema, and sometimes perirectal fissuring. In a hospital-based study performed by Mostafa et al. [34], in Egypt on 150 children with perianal dermatitis, hemolytic streptococci was found in 35.3% of the cases, half of which were of the group A hemolytic strain (17.3%) and half of which were nongroup A (18%).

The age range of patients in the current study was between 8 and 23 months (a mean age of 15.01 ± 3.26 months). This was higher than the age of diaper dermatitis observed by Jordan et al., [2] which was 9–12 months. This difference may be due to our inclusion of perianal inflammation rather than all cases of diaper dermatitis and inclusion of recurrent rather than nonrecurrent ones.

In our study, slight male preponderance was found (52.38%). This was similar to Kränke et al. [4] who reported a male preponderance of 57.1%.

Age at onset of first episode of dermatitis ranged between 4 and 20 months (a mean of 11.2 ± 3.7 months). Number of recurrences ranged between 2 and 6 (a mean of 3.6 ± 1.3 attacks). Duration of the last attack ranged between 12 and 30 days (a mean of 20.1 ± 3.5 days). The duration of dermatitis from first diagnosis till time of definitive diagnosis ranged between 3–7 months (a mean of 3.92 ± 2.81 months). This seems lower than that reported by Kränke et al. [4] who found that half of the patients (51.6%) had complaints for more than 12 months.

From the clinical point of view, vomiting, abdominal distension and atopic diseases were more commonly encountered in the group of cow's milk allergy (83.3%, 70%, and 76.7%, resp.) compared to other patients (24.24%, 18.18%, and 6%, resp.). They can be considered clinical predictors of the possibility of cow's milk allergy.

The higher frequency of atopic diseases in CMA group can be supported by finding of Isolauri et al. [35] that a history of atopy is more common in children with cow's milk protein allergy. Yet, in another study by Doganci and Cengizlier [26], only 2% of children with CMA had atopic diseases. This difference can be explained by difference in pathogenic basis of CMA disease in different population. Doganci and Cengizlier included constipation only. This may be related to the nature of the disease whether it was IgE or non-IgE mediated.

No significant difference was found between children with CMA and other causes as regards family history of atopy (*P* = 0.9801). Matching with this result, Doganci and Cengizlier [26] found that none of children with CMA had history of familial atopic diseases. On the contrary, Iacono et al. [27] demonstrated a higher frequency of family histories of atopy among patients with cow's milk allergy related chronic constipation. These discrepant results may reflect either a nonunified way of diagnosis of cow's milk allergy or difference in rate of consanguinity in various countries.

Diarrhea and constipation were not different between both groups (*P* > 0.05). Perianal ulceration and anal fissuring were more commonly encountered with cow's milk allergy group (*P* < 0.05).

A common cause in our study was lactose intolerance. Radlović et al. [25] found that strict diet eliminating gluten as well as contemporary elimination of lactose for only 2-3 weeks in the patient's nutrition helped to improve the perianal erythema noticed in patients with gluten sensitive enteropathy.

In the present work, small bowel bacterial overgrowth was associated with perianal bacterial dermatitis. We cannot set an answer whether the problem was only bacterial infection from the start or initiated a state of lactose intolerance that triggered the dermatitis. Zhao et al. [36] concluded that small intestinal bacterial overgrowth increases the likelihood of lactose intolerance.

On multiregression analysis it was found that recurrent vomiting, other atopic manifestations, anal fissures, perianal ulcers as well as presence of blood and/or mucus in stool are the most important predictors of underlying cow's milk allergy as a cause of recurrent perianal dermatitis. The age of presentation was not a significant determinant of cow's milk allergy as other etiologies of the current problem are common below 1 year age similar to cow's milk allergy. 

We can conclude that recurrent perianal dermatitis is a multifactorial problem with cow's milk allergy being the most common cause. Recurrent vomiting, other atopic features, fissures and ulcers as well as presence of mucus or blood in stool are significant predictors of this etiology in such a clinical context.

## Figures and Tables

**Table 1:** Comparison of qualitative clinical and laboratory data between CMA group and other causes.

	CMA (30)	Others (33)	*X* ^2^	*P*
FH of erythema	11	8	1.15	0.2832
FH of atopy	11	12	0.0001	0.9801
Vomiting	25	8	22.00	0.0001
Diarrhea	27	28	0.38	0.5397
Constipation	3	1	1.28	0.2572
Abdominal distension	21	6	17.23	<0.0001
Atopic features in patients	23	2	32.73	<0.0001
Presence of ulcers	26	16	10.31	0.0013
Presence of satellites	1	19	21.34	<0.0001
Presence of anal fissures	24	3	32.26	<0.000
Presence of macroscopic pus	28	12	22.00	<0.0001
Presence of macroscopic blood	27	5	35.22	<0.0001
Microscopic pus cells	22	8	15.18	<0.0001
Microscopic RBCS	28	6	35.73	<0.0001
Stool reducing substances	1	11	9.17	0.0025
Pathogenic organisms	2	13	9.28	0.0023

**Table 2:** Comparison of quantitative clinical and laboratory data between CMA group and other causes.

	CMA	Others	*t*	P
Age	15.10 ± 0.75	15.09 ± 2.79	0.01	0.991
Age of onset	11.10 ± 4.47	11.24 ± 2.94	−0.15	0.881
RAST	4.26 ± 1.28	0.19 ± 0.06	18.15	<0.0001
Size of erythema	2.69 ± 0.59	4.03 ± 1.20	−5.50	<0.0001
Duration of last attack	20.33 ± 3.58	20.00 ± 3.54	0.37	0.712
Recurrence rate of erythema	3.56 ± 1.54	3.66 ± 1.16	−0.29	0.772
pH of stool	6.68 ± 0.61	5.68 ± 1.87	2.782	0.007

**Table 3:** Regression analysis (logistic regression) of predictors of CMA as an underlying cause of perianal dermatitis.

	Score	*P*
Age of onset of first lesion	0.165	0.684
Age at presentation	0.135	0.713
Family history of atopy	0.000	1.000
Recurrent vomiting	9.000	0.003
Abdominal distension	3.571	0.059
Atopic associations	16.026	<0.0001
Ulcers	8.048	0.005
Satellites	5.818	0.016
Fissures	16.000	<0.0001
Mucus	15.696	<0.0001
Blood	25.138	<0.0001
